# Supplementary material for: Transcriptomic Insights into Mechanisms of Early Seed Maturation in the Garden Pea (Pisum sativum L.)
Source: Cells. 2020 Mar 23;9(3):779. doi: 10.3390/cells9030779 (PMC7140803; doi:10.3390/cells9030779)

Sprint−2(20) vs Zhewa

Global Summary

%DE = 0.83

genes with fdr < 0.2 = 1673 (852 + / 821 −)

genes with fdr < 0.1 = 1484 (740 + / 744 −)

genes with fdr < 0.05 = 1285 (633 + / 652 −)

genes with fdr < 0.01 = 884 (407 + / 477 −)

genes in genesets = 0

<FC> = 0

<t−score> = 1414.42

<p−value> = 0

<fdr> = 0.17

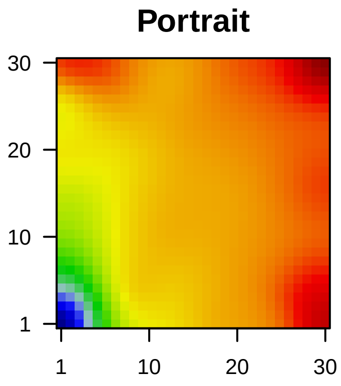

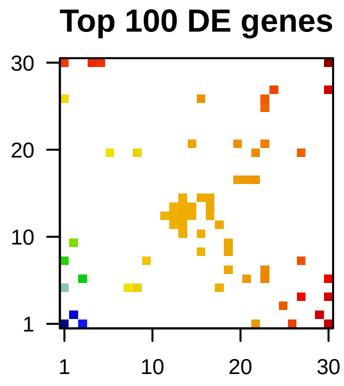

Global Genelist

Rank	ID	log(FC)	p−value	fdr / Metagene	Description
1	TRINITY_DN4	6.62	0e+00	0.00000014 x 30	
2	TRINITY_DN2	−0.15	1e−10	0.00000014 x 1	
3	TRINITY_DN3	−0.01	2e−10	0.00000019 x 14	
4	TRINITY_DN9	−0.01	7e−10	0.00000023 x 14	
5	TRINITY_DN4	0.11	2e−09	0.00000023 x 27	
6	TRINITY_DN2	−1.86	2e−09	0.00000023 x 1	
7	TRINITY_DN2	−0.01	3e−09	0.00000026 x 26	
8	TRINITY_DN2	−1.33	3e−09	0.00000040 x 1	
9	TRINITY_DN9	−0.01	5e−09	0.00000043 x 12	
10	TRINITY_DN5	−0.07	6e−09	0.00000040 x 20	
11	TRINITY_DN1	0.01	9e−09	0.00000045 x 21	
12	TRINITY_DN1	−0.01	1e−08	0.00000043 x 12	
13	TRINITY_DN1	−0.01	1e−08	0.00000044 x 11	
14	TRINITY_DN9	−0.01	1e−08	0.00000043 x 12	
15	TRINITY_DN1	−0.01	1e−08	0.00000043 x 12	
16	TRINITY_DN1	−0.01	1e−08	0.00000043 x 12	
17	TRINITY_DN1	−0.01	1e−08	0.00000219 x 12	
18	TRINITY_DN4	−0.03	3e−08	0.00000289 x 5	
19	TRINITY_DN1	−0.01	3e−08	0.00000214 x 13	
20	TRINITY_DN1	−0.01	3e−08	0.00000219 x 13	

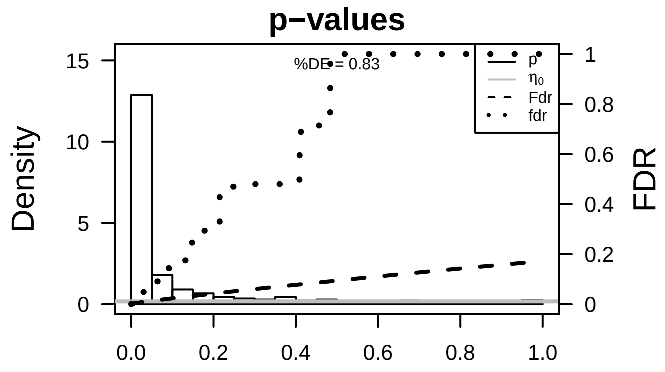

Supplement: Supplementary file 1 [file cells-09-00779-s001.zip › Supplementary materials/FileS4.zip!/FileS3/TFs - Results/Summary Sheets - Differences/Reports/Sprint.2.20..vs.Zhewan.1.10..pdf]
